# Managing the Heterogeneity of Mesenchymal Stem Cells for Cartilage Regenerative Therapy: A Review

**DOI:** 10.3390/bioengineering10030355

**Published:** 2023-03-13

**Authors:** Doreen Goh, Yanmeng Yang, Eng Hin Lee, James Hoi Po Hui, Zheng Yang

**Affiliations:** 1 Department of Orthopaedic Surgery, Yong Loo Lin School of Medicine, National University of Singapore, 1E Kent Ridge Road, NUHS Tower block Level 11, Singapore 119288, Singapore; 2 NUS Tissue Engineering Program, Life Sciences Institute, National University of Singapore, 27 Medical Drive, DSO (Kent Ridge) Building, Level 4, Singapore 11751, Singapore; 3 Critical Analytics for Manufacturing Personalised-Medicine, Singapore-MIT Alliance for Research and Technology, Singapore 138602, Singapore

**Keywords:** mesenchymal stem cells, heterogeneity, cartilage regeneration, stem cell therapy

## Abstract

Articular cartilage defects commonly result from trauma and are associated with significant morbidity. Since cartilage is an avascular, aneural, and alymphatic tissue with a poor intrinsic healing ability, the regeneration of functional hyaline cartilage remains a difficult clinical problem. Mesenchymal stem cells (MSCs) are multipotent cells with multilineage differentiation potential, including the ability to differentiate into chondrocytes. Due to their availability and ease of ex vivo expansion, clinicians are increasingly applying MSCs in the treatment of cartilage lesions. However, despite encouraging pre-clinical and clinical data, inconsistencies in MSC proliferative and chondrogenic potential depending on donor, tissue source, cell subset, culture conditions, and handling techniques remain a key barrier to widespread clinical application of MSC therapy in cartilage regeneration. In this review, we highlight the strategies to manage the heterogeneity of MSCs ex vivo for more effective cartilage repair, including reducing the MSC culture expansion period, and selecting MSCs with higher chondrogenic potential through specific genetic markers, surface markers, and biophysical attributes. The accomplishment of a less heterogeneous population of culture-expanded MSCs may improve the scalability, reproducibility, and standardisation of MSC therapy for clinical application in cartilage regeneration.

## 1. Articular Cartilage Injury and Management

Articular cartilage is a highly specialised tissue found in synovial joints at the articulating surfaces of bones. It has a low density of chondrocytes, which produce and maintain a rich extracellular matrix (ECM) comprising collagens, proteoglycans, water, and ions [1]. The dense collagen network contributes to tensile strength and shear stiffness of articular cartilage [2]. The high proteoglycan content in cartilage enables articular cartilage to withstand compressive forces and allows for weight-bearing. With the secretion of lubricin by superficial zone chondrocytes, cartilage is coated by a lubricating layer and has a low coefficient of friction, providing a smooth surface for uninterrupted joint movement [3].

However, repeated high-intensity mechanical overloading and acute trauma can cause cartilage injury. Given its avascular, aneural, and alymphatic nature [4], articular cartilage has a limited intrinsic regenerative capacity and does not recover readily from injury. This is exacerbated by the low density of chondrocytes in articular cartilage [5] and the high density of cartilage ECM, which impedes the migration of local chondrocytes to the injury site [6]. If cartilage injury is allowed to progress, changes in load bearing and the release of inflammatory mediators [7] can result in cumulative cartilage degeneration with secondary synovitis, subchondral bone remodelling, and contractions in the surrounding ligaments, joint capsule, and muscles [8]. This predisposes the patient to developing osteoarthritis.

Current management of cartilage injury includes surgical debridement of the joint, bone marrow stimulation, and transplantation approaches. In surgical debridement, loose bodies and calcified cartilage are removed from the joint to avoid further deterioration and fragmentation of the articular surface [9]. Bone marrow stimulation techniques, such as drilling, microfracture, and abrasion chondroplasty, induce the migration of bone marrow cells into the cartilage defect by mechanically penetrating the underlying subchondral bone. A fibrin clot, containing extravasated bone marrow mesenchymal stem cells (BM-MSCs), forms in the defect. Mesenchymal stem cells (MSCs) then participate in cartilage regeneration [10]. However, repair after bone marrow stimulation mostly results in the formation of biomechanically inferior fibrocartilage containing type I collagen, instead of the desired hyaline cartilage with type II collagen [11]. The fibrocartilage repair tissue is poorly integrated into the native cartilage and deteriorates over time [12], leading to declining treatment effectiveness in long-term studies [13,14]. Bone marrow stimulation techniques also disrupt the integrity of the subchondral bone, leading to instability and the formation of subchondral cysts and osteophytes [15].

Alternatively, surgeons can consider transplantation techniques for the management of articular cartilage defects. Osteochondral autograft transfers, either with the osteochondral autograft transfer system (OATS), or Mosaicplasty, are indicated in smaller defects less than 4 cm^2^ [16]. They involve the transplantation of healthy osteochondral tissue from non-weight-bearing areas of the joint into the defect. However, these techniques are limited by donor site morbidity and the scarce amount of donor tissue that can be harvested. Non-weight-bearing donor cartilage may also have differences in mechanical properties and depth [17]. In larger defects, osteochondral allografting can be applied instead, which involves the transplantation of osteochondral tissue from a cadaveric source. Osteochondral allografting avoids donor-site morbidity and limitations in tissue size and structure. However, allografts are associated with high cost, and the risk of tissue rejection and disease transmission [18].

Autologous chondrocyte implantation (ACI) is the first application of cell therapy in orthopaedics. ACI is suited for defect sizes larger than 3 to 4 cm^2^ [19]. First-generation ACI is a two-stage procedure that consists of cartilage harvesting from the joint, in vitro isolation and expansion of chondrocytes, and subsequent reintroduction of chondrocytes into the chondral defect under a periosteal patch [20]. However, the periosteal patch is prone to hypertrophy, with more than 20% of patients requiring repeat arthroscopy with debridement. In second generation ACI, a collagen membrane replaces the periosteal patch for covering the defect. In third generation ACI, tissue engineering principles are applied to incorporate chondrocytes directly onto a three-dimensional (3D) scaffold for implantation. The scaffold allows for better graft stability and reduces dedifferentiation of chondrocytes, generating more hyaline-like cartilage [21,22]. Also known as matrix-induced autologous cartilage implantation (M-ACI), this technique has led to improved clinical outcomes in patients compared to microfracture [23].

Despite the promising clinical outcomes of cell therapy approaches, the use of autologous chondrocytes requires tissue harvesting from the non-weight-bearing areas of knee cartilage, resulting in donor site morbidity. Chondrocytes are also scarce, with only 10 cells available in every 0.22 mm^2^ of human knee articular cartilage [24], necessitating the extensive ex vivo expansion of limited chondrocytes after isolation from patient tissue. Yet, chondrocytes that are cultured for prolonged periods are prone to dedifferentiation, during which cells become fibroblastic with reduced expression of type II collagen, generating neo-cartilage of lower functional efficacy, with mechanically inferior ECM [25]. Clinicians have, hence, explored the use of stem cells, especially adult MSCs, as an alternative cell source for the treatment of cartilage lesions.

## 2. Mesenchymal Stem Cells (MSCs) for Cartilage Regeneration

MSCs are multipotent cells with a multilineage differentiation potential, including the ability to differentiate into chondrocytes and generate ECM containing type II collagen. The use of MSCs as an alternative cell source for cartilage regeneration confers numerous advantages. Firstly, autologous MSCs can be harvested relatively easily through less invasive means, without injuring existing healthy cartilage [26]. Various tissue sources are available for MSC harvest, including bone marrow, adipose tissue, umbilical cord blood, and synovial fluid [27,28]. Unlike chondrocytes, MSCs can undergo expansion in vitro without losing their phenotype through dedifferentiation [29]. Allogenic cells can also be used in tissue engineering, as MSCs have been shown to be immune-evasive [30].

As such, many pre-clinical studies and clinical trials have been conducted on the use of MSCs for cartilage regeneration [31]. In vivo, intra-articular implantation of MSCs has led to enhanced cartilage repair in both small and large animal models [32]. Clinically, seven years of extended follow up, after implantation of MSCs encapsulated within a hyaluronic acid hydrogel, have found MSCs to be safe and efficacious for the treatment of cartilage defects [33]. As an adjuvant treatment, a large multicentre prospective randomised clinical trial has recently found that the intra-articular injection of BM-MSCs after microfracture led to improved repair, and a trend towards improved clinical outcomes in articular cartilage injuries of the knee [34]. A single-stage cartilage repair procedure, employing allogenic MSCs and autologous chondrons in fibrin glue, has similarly reported good five-year safety outcomes and led to clinical improvement [35].

In addition to being capable of chondrogenic differentiation, MSCs exert trophic effects on the surrounding chondrocytes, secreting bioactive molecules that promote ECM formation [36]. The therapeutic potential of the MSC secretome has led to an increased focus on cell-free strategies to facilitate cartilage repair. A systematic review of pre-clinical research reports that MSC exosomes are effective in the treatment of articular cartilage injury, resulting in hyaline-like neocartilage formation, improved integration with native cartilage, and increased ECM deposition in the defect site [37]. Exosomes secreted by MSCs enhance cartilage regeneration through multiple pathways, including the activation of AKT and ERK signalling to promote chondrocyte proliferation [38]. CD73 found in MSC exosomes also initiate tissue repair by hydrolysing the post-injury extracellular pro-apoptotic ATP signal into the pro-survival adenosine signal, while exosomal miRNAs are involved in MSC chondrogenesis and matrix synthesis [39]. MSCs also promote a regenerative milieu in injured tissue, increasing the infiltration of regenerative M2 macrophages and inhibiting pro-inflammatory cytokines [38,40]. The MSC secretome further increases the anti-inflammatory IL-10 and TGF-β1 levels, and induces regulatory T cells through CCL18 signalling [39,41]. miRNAs found in MSC exosomes contribute to the immunomodulatory role of MSCs [42]. In an in vivo porcine model, the injection of MSC exosomes and hyaluronic acid has led to improved morphological, histological, and functional recovery after 4 months [43].

## 3. Heterogeneity of MSCs

Despite encouraging pre-clinical and clinical outcomes, inconsistencies in MSC characteristics remain a key barrier to widespread clinical application of MSC therapy in cartilage regeneration. The high degree of variability in MSC stemness and chondrogenic potential poses a challenge to the scalability, reproducibility, and standardisation of therapeutic potency [44,45]. The characterisation of MSC exosomal mechanisms of action remains limited, partly due to MSC heterogeneity. This restricts further research on MSC secretome potency, and may limit the future clinical application and commercialisation of MSC exosome therapy [46].

MSC heterogeneity can occur at multiple levels, depending on the donor, MSC tissue source, cell subset, culture conditions, and handling techniques (Table 1). There is a large degree of inter-individual variation in MSC quality, even when isolated from the same tissue source [47]. As donor age increases, MSC immunomodulation, proliferation, and differentiation potential decreases [48]. Compared to older donors, BM-MSCs from young donors have improved glycosaminoglycan deposition and increased expression of the chondrogenic markers *SOX9*, *COL2A1*, and *ACAN* [49]. Donor sex affects MSC properties as well. MSCs from young female donors have been shown to exhibit a shorter population doubling time and increased colony formation [50]. MSCs from donors with diseases such as osteoarthritis and osteoporosis demonstrate lower chondrogenic potential and proliferative ability, with a reduced expression of MSC surface marker CD73 [51,52]. On a genomic level, BM-MSCs from donors with a genomic deletion of glutathione S-transferase theta 1 (*GSTT1*) demonstrate increased scalability, with improved clonogenic and proliferative capabilities, while maintaining a robust multilineage differentiation potential [53].

MSCs are commonly derived from the bone marrow, adipose tissue, umbilical cord blood, and synovium. On an intra-individual level, cells from different tissue sources demonstrate heterogeneity in MSC characteristics. Compared to adipose-derived MSCs (AD-MSCs), BM-MSCs from the same donor exhibited a better chondrogenic potential in vitro and in vivo [54,55]. The seeding of BM-MSCs in commercially available scaffolds has led to better ECM deposition in vitro than seeding with AD-MSCs [56]; the seeding of BM-MSCs in a platelet-rich plasma bioactive scaffold has led to improved macroscopic, histological, and immunohistochemical characteristics compared to AD-MSCs in an in vivo rabbit model [57]. Although inferior in chondrogenic potential, AD-MSCs may possess an improved proliferative ability compared to BM-MSCs [58]. Umbilical cord blood-derived MSCs (UCB-MSCs) have a higher population doubling number than BM-MSCs [27]. However, compared to BM-MSCs, UCB-MSCs are reported to produce ECM of lower quantity after chondrogenesis [59]. Synovium membrane-derived MSCs (SM-MSCs) are another explored population of MSCs. Previous studies have found that SM-MSCs demonstrate an increased proliferative ability and enhanced chondrogenic potential compared to donor-matched BM-MSCs and AD-MSCs [28]. The implantation of SM-MSCs in human knee articular cartilage defects has resulted in the production of hyaline cartilaginous tissue and improved clinical outcomes [60].

Variation further exists between MSCs of a similar tissue source that are isolated from different anatomical locations. For example, a higher chondrogenic potential has been demonstrated by BM-MSCs extracted from the iliac crest and vertebral body compared to cells derived from the femoral head, possibly due to an increased involvement of the femoral head in disease processes [61]. BM-MSCs collected from the proximal femur trabeculae, through rasping, display an increased chondrogenic potential compared to BM-MSCs harvested from the main marrow compartment of the same patients through BM aspiration [62]. Among the AD-MSCs derived from various anatomical locations, the AD-MSCs from subcutaneous tissue show increased proliferative ability [63]. However, a donor-matched study has found that AD-MSCs derived from the infrapatellar fat pad demonstrate increased glycosaminoglycan production and upregulation of the chondrogenic genes *ACAN* and *COL2A1*, indicating an increased chondrogenic potential compared to AD-MSCs from subcutaneous fat [64].

During in vitro culture, MSCs form different single cell-derived clonal subpopulations within the same MSC preparation. These subpopulations vary in morphology, transcriptome, proliferative potential, and functional chondrogenic differentiation potential [65,66,67]. In fact, the MSC subpopulation phenotype may have a larger role in influencing the MSC chondrogenic potential and secretome, compared to inter-individual heterogeneity in donor age or gender [50]. Heterogeneity in MSCs is present even at an intra-colony level, despite cells being supposedly derived from a single MSC and cultured under similar conditions. Within the same colony, cell-to-cell variation exists in the morphology and gene expression [68]. Single cells differentially express immunomodulatory and chondrogenic genes, and cells with diverse properties can ultimately result from a once-homogenous colony [69,70,71].

## 4. Managing the Heterogeneity of MSCs for More Effective Cartilage Regeneration

**Figure 1:**
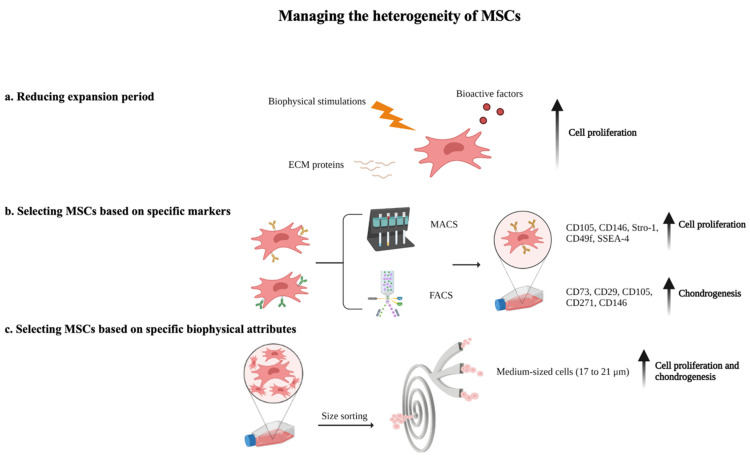
Managing the heterogeneity of MSCs. The heterogeneity of MSCs can be managed ex vivo through (**a**) reducing MSC culture expansion with the addition of bioactive factors, extracellular matrix proteins, and biophysical stimulation, (**b**) selecting MSCs with a higher chondrogenic potential based on specific surface markers, and (**c**) selecting MSCs with a higher chondrogenic potential based on specific biophysical attributes.

In addition to selecting the appropriate donor and MSC tissue source, this section elaborates on other strategies to manage the heterogeneity of MSCs ex vivo for more effective cartilage repair, namely, through reducing the MSC culture expansion period, and selecting MSCs with a higher chondrogenic potential, through specific surface markers and biophysical attributes (Figure 1).

### 4.1. Reducing Expansion Period

The aforementioned diversity in MSC subpopulations emerges and becomes amplified during culture expansion, with single colonies ultimately giving way to a heterogenous mix of cells characterised by varying potencies [72,73]. Extensive passaging in vitro also results in a decreased MSC migration potential and differentiation potential, leading to the production of less cartilaginous tissue [74]. MSCs in long-term culture produce increased β-galactosidase levels compared to MSCs from aged donors, indicating that culture length may have a greater impact on MSC senescence than donor age [75]. After extended culture expansion, AD-MSCs also demonstrate an increased production of proinflammatory cytokines and a decreased production of anti-inflammatory mediators [76]. Thus, reducing the expansion period of MSCs could improve MSC homogeneity and quality for cartilage repair.

To reduce the MSC expansion period, MSC proliferation may be enhanced with the addition of bioactive factors. Growth factors, such as fibroblast growth factor 2 (FGF2), have been shown to improve MSC proliferative capabilities in vitro, reducing the population doubling time and resulting in differential expression of proliferation-related genes [77]. After four passages, FGF2 treatment increases cell yield by 256-fold without affecting the MSC chondrogenic potential [78]. Binding of the FGF receptor may activate signalling pathways, such as the Ras/Erk pathway, to increase cell proliferation downstream [79,80]. Supplementation with FGF4 also leads to a halving of the MSC population doubling time without affecting MSC pluripotency. As a ligand for the FGF2 receptor, FGF4 may exert its stimulatory effects on MSC proliferation through similar signalling pathways [81]. Heparan sulfate is another activator of FGF2 signalling through its affinity for FGF2 and the FGF2 receptor [82]. Heparan sulfate increases endogenous FGF2 production, and stabilises FGF2 to maximise the effects of FGF2 supplementation [83,84]. The addition of heparan sulfate increases MSC colony formation and growth, increasing MSC expansion by up to 13-fold while maintaining MSC multipotency [85].

Platelet-derived growth factor-BB (PDGF-BB) stimulates MSC self-renewal through the PI3K/Akt pathway [86], increasing the expression of membrane-tethered matrix metalloproteinase MT1-MMP to enhance MSC proliferation [87]. PDGF-BB exerts a dose-dependent effect on MSC numbers, increasing MSC cell count by 45% at 5 nM, and 81% at 25 nM [88]. A potent mitogen, PDGF-BB upregulates the genes associated with proliferation without promoting senescence [89].

Other bioactive additives to consider include epidermal growth factor (EGF) and insulin-like growth factor (IGF). EGF increases colony-forming units and improves MSC proliferation in vitro by 30% [90], activating β-catenin to reduce cell cycle pathway suppression [91]. Elevated autocrine IGF-1 levels also enhance MSC proliferation and the number of cell doublings while suppressing apoptosis [92]. IGF-1 supplementation stimulates proliferation in a dose-dependent manner through both the IGF-1 receptor and insulin receptor [93].

In addition to growth factors, ECM proteins have a role to play in MSC proliferation. Tropoelastin is an ECM component which interacts with the cell surface integrins αvβ3 and αvβ5. Tropoelastin supplementation stimulates MSC proliferation at a level similar to IGF-1 or FGF-2 supplementation while maintaining the MSC phenotype, and may be an attractive cost-effective alternative to the use of serum and growth factors [94]. MSCs cultured with basement membrane ECM proteins further demonstrate a 250-fold increase in proliferation capabilities, resulting in increased colony numbers and size while retaining MSC multipotency [95]. Coating culture flasks with type I collagen significantly increases MSC proliferation, boosting cell attachment and reducing cell death [96]. However, effects on the subsequent MSC chondrogenic potential have not been investigated. ECM produced from stromal cells increases cell proliferation in a three-dimensional scaffold, and enhances MSC clonogenicity while maintaining MSC stemness [97].

Lastly, biophysical stimulation through low intensity vibration, electromagnetic field exposure, and electrical stimulation may contribute to improved MSC proliferation during culture expansion. Low intensity vibration increases MSC doubling by 28%, and reduces senescence-associated β-galactosidase levels by 28% after 60 passages in vitro [98]. Loss of the MSC proliferative ability can also be reversed with the application of low intensity vibration [99]. Electromagnetic field exposure of suspended MSCs increases MSC numbers by 2.41-fold [100], possibly due to the induction of a shorter cell cycle [101]. Electrical stimulation of MSCs at 448 kHz induces cell cycle progression through ERK1/2 upregulation, enhancing MSC proliferation by 38% without affecting subsequent MSC differentiation [102].

### 4.2. Selecting MSCs Based on Specific Markers

The expression of specific cell surface markers has been linked to an increased cartilage regeneration potential of MSC subpopulations [48] (Table 2). Screening MSCs for potency allows for the selective expansion of MSC subpopulations with homogenous and desired characteristics, increasing the applicability of MSC therapy. Selection may be performed with techniques such as magnetic-activated cell sorting (MACS) or fluorescence-activated cell sorting (FACS) [103], and with a combination of markers to potentially improve screening specificity [104].

The MSC surface markers specified by the International Society for Cell and Gene Therapy are CD73, CD90, and CD105 [44,105]. CD73^+^ MSCs possess a good chondrogenic potential, with high levels of *COL2* and *ACAN* expression during chondrogenesis [106]. Meanwhile, CD105^+^ MSCs demonstrate increased proliferation, improved colony formation, and an enhanced chondrogenic potential in vitro with increased expression of *SOX9*, *COL2*, and *ACAN* [107,108]. Subpopulations of AD-MSCs expressing CD29 and CD105 show an increased chondrogenic potential, witha higher expression of the chondrogenic marker *COL2* [109].

A member of the tumour necrosis factor receptor superfamily, CD271, is a cell surface marker that establishes MSC proliferation and differentiation capacity [110]. Compared to CD73^+^ MSCs, CD271^+^ MSCs have an increased chondrogenic potential [106]. CD271^+^ BM-MSCs display improved chondrogenesis in vitro and in vivo compared to control MSCs, and a reduced volume of CD271^+^ BM-MSCs is needed to produce a similar cartilage graft compared to unfiltered multiclonal MSC preparations [111,112]. CD271^+^ MSCs improve osteochondral defect healing, and maintain low angiogenesis while retaining trilineage multipotency [113]. In addition to demonstrating upregulation of the genes associated with ECM production and cell adhesion, CD271^+^ MSCs demonstrate downregulation of the genes associated with inflammation [114].

CD146 is a transmembrane glycoprotein expressed in MSCs but not in fibroblasts [115]. CD146 expression decreases as the MSC passage number increases, coinciding with an increase in population doubling time [116]. CD146^+^ MSCs produce increased glycosaminoglycans after chondrogenesis [117] and demonstrate an increased chondrogenic potential [118]. In a three-dimensional scaffold, CD146^+^ MSCs demonstrate spontaneous chondrogenesis [119], while intra-articular implantation of CD146^+^ MSCs has led to chondroprotective effects in inflammatory environments [120]. A magnetically-sorted CD146^+^ MSC population has also shown improved cell migration compared to a heterogenous cell population [121]. Compared to unsorted AD-MSCs, CD146^+^ MSCs promote long-term cartilage repair in vivo and produce less inflammation [122].

Stro-1 is another widely-employed MSC marker [123]. Stro-1 expression is associated with increased proliferation and differentiation capacity [124], along with increased immunosuppression and homing capabilities of MSCs [125]. Meanwhile, CD49f is a member of the adhesion molecular family that is also involved in the maintenance of MSC stemness, regulating MSC proliferation and differentiation through the PI3K/AKT signalling pathway [126]. CD49f^+^ MSCs have improved clonogenicity, adhesion, migration, and anti-apoptotic properties compared to unsorted MSCs, with MSCs demonstrating reduced CD49f expression as the passage number increases [127,128]. The glycolipid SSEA-4 may be another surface marker of interest. Compared to unsorted MSCs, SSEA-4^+^ MSCs exhibit improved growth and multipotency [129,130]. Increased SSEA-4 expression may also be associated with increased chondrogenic potential [131]. Although these surface markers have not been well-studied in the context of chondrogenesis and cartilage repair, the superior MSC characteristics of cells expressing Stro-1, CD49, and SSEA-4 may render these specific markers an attractive target in MSC selection for more effective clinical application.

Alternatively, genomic biomarkers, such as *GSTT1*, may be used for the selection of MSC donors pre-harvest. Transcriptomic analyses have revealed that homozygous negative *GSTT1* MSCs demonstrate increased scalability and potency, possibly due to increased protection from DNA damage [53]. Diagnostic kits to identify prospective donors are being developed, and may reduce unnecessary MSC harvesting from donors [132]. Other hypothesised genetic biomarkers, which may be validated in future studies, include the single-nucleotide polymorphism rs144383 in *GDF5*, which is strongly associated with osteoarthritis [133].

With the high cell-to-cell variability present within a single MSC colony, single-cell profiling techniques may become increasingly important to identify heterogeneity within MSC subpopulations, and establish more selective biomarkers for isolating MSC subpopulations capable of more effective cartilage repair. Single-cell RNA sequencing (scRNA-seq) allows for the ongoing identification of functional MSC subpopulations through clustering [134]. Surface marker expression and the subsequent heterogeneity during chondrogenesis can be quantified to identify cell subpopulations that are less heterogenous and more chondrogenic [135]. At a genomic level, techniques such as multiple displacement amplification (MDA) and multiple annealing and looping-based amplification cycles (MALBAC) may be employed, while single-cell mass spectrometry may be used to explore cellular heterogeneity at a proteomic level [136,137].

**Table 2:** A summary of studies that highlight specific MSC markers and their associated phenotype in cartilage regenerative therapy.

Specific MSC Markers	Results	Reference
CD73+	High levels of *COL2* and *ACAN* expression during chondrogenic differentiation, with stable levels of COL1, COLX, and MMP13.	[106]
CD105+	Increased proliferation and improved colony formation. Enhanced chondrogenic potential in vitro with increased expression of *SOX9*, *COL2*, and *ACAN*.	[107,108]
CD271+	Improved chondrogenic differentiation with a higher expression of *Runx2* and *COL2*. Upregulation of genes associated with ECM production and cell adhesion; downregulation of genes associated with inflammation. Improved osteochondral defect healing while maintaining low angiogenesis in an athymic rat model.	[106,113,114]
CD146+	Increased glycosaminoglycan production after chondrogenesis. Improved chondrogenic potential and cell migration. Chondroprotective effects during intra-articular implantation. Promoted long-term cartilage repair in a rat osteochondral defect model and demonstrated immunomodulation.	[117,118,119,120,121,122]
Stro-1+	Increased proliferation and differentiation capacity. Increased immunosuppression and homing capabilities.	[124,125]
CD49f+	Improved clonogenicity, adhesion, migration, and anti-apoptotic properties.	[127]
SSEA-4	Improved growth and multipotency. Increased chondrogenicity.	[129,130,131]
*GSTT1*	Homozygous negative *GSTT1* MSCs demonstrate increased scalability and potency.	[53]

### 4.3. Selecting MSCs Based on Specific Biophysical Attributes

MSCs develop variation in morphology and physical properties with prolonged in vitro expansion, with multivariate biophysical analysis of culture-expanded MSCs further demonstrating a correlation between cell size, stiffness, nuclear membrane fluctuations, MSC biomolecular markers, and functionality [138,139]. High-throughput cell separation techniques are being developed for the selection of MSCs based on these properties. Multiparameter deformability cytometry classifies cells according to multiple biophysical characteristics, such as size, deformability, and morphology, through microfluidic inertial focusing, hydrodynamic stretching of cells, and high-speed video recording [140]. Since MSC differentiation results in a reorganisation of lamin A/C and an increase in heterochromatin levels [141], MSC stiffness is also directly correlated with the extent of chondrogenic differentiation [142]. The combination of small cell size, low cell stiffness, and high nuclear membrane fluctuations is indicative of high MSC clonogenicity and multipotency, and future cell isolation techniques may select for this subpopulation of MSCs [138]. The corresponding surface biomarkers of the cell mechanophenotype may also be employed for MSC sorting. For example, CD44 expression is strongly inversely related with cell elasticity, and MSC isolation based on cell stiffness may employ CD44 expression levels as a surrogate marker [143]. Alternatively, cell separation based on cell deformability using a deterministic lateral displacement device, such as those employed in isolating immune cells from whole blood cells, may be used to segregate MSC based on stiffness [144].

Functional differences in the capacity for cartilage repair are present based purely on MSC size. Using a high-throughput microfluidic label-free technology, medium-sized cells, 17 to 21 μm in size, have been demonstrated to possess the highest proliferation rate and chondrogenic potential. Selected MSCs of this size continue to highly express *COL2* and *ACAN* after 10 passages, prolonging the MSC chondrogenic potential in vitro [145]. In addition, the secretome of MSCs within this size range promotes chondrogenesis, while the secretome of larger MSCs, more than 21 μm in size, promotes osteogenesis and adipogenesis [139,146]. Further, the secretome of larger MSCs has been found to further induce cellular senescence, indicating the negative effect of MSC heterogeneity during in vitro expansion. A selective MSC culture approach, that excludes MSCs larger than 21 μm and smaller than 17 μm at every passage, may select for MSCs with an improved proliferation and chondrogenic potential compared to a conventional MSC expansion approach [145,146].

Another potentially relevant biophysical attribute is cell and nuclei shape. MSCs take on a rounded phenotype at the start of chondrogenesis, and rounded MSCs in early lineage commitment have been found to upregulate chondrogenic genes, committing to chondrogenesis [147]. This may be associated with the MT1-MMP control of MSC commitment through ECM remodelling, in which MT1-MMP activates β1-integrin for downstream signalling through regulating MSC shape [148]. MSCs with a rounded nuclei have also been found to increase the expression of *RUNX2*, *SOX9*, and *ACAN* [149].

## 5. Conclusions

Articular cartilage injury severely impacts patients’ quality of life, driving a market for cartilage regeneration products that was worth USD 852 million in 2021 [150]. While MSC therapy has the potential to improve cartilage repair, MSCs are highly heterogenous, and inconsistencies in MSC quality prevent scalability, reproducibility, and standardisation for clinical application. Hence, large-scale clinical trials employing MSC therapy for cartilage regeneration have not been completed [151]. To hasten the advancement of MSC therapy, numerous companies have developed proprietary MSC-specific optimised expansion media that are serum- and xeno-free for the reliable expansion of MSCs. The identification of suitable genetic markers may further improve the selection of donors for high-performance MSCs in allogenic therapy. In addition, the employment of optimal biophysical attributes for the selection of desirable chondrogenic MSCs may facilitate the delivery of a more functionally consistent MSC phenotype, thus enabling the translation of MSC therapy to the clinic for improved cartilage regeneration.

## Figures and Tables

**Table 1:** A summary of studies that highlight sources of MSC heterogeneity and their corresponding outcomes in cartilage regenerative therapy.

Source of Heterogeneity	Study Details	Results	Reference
Donor	BM-MSCs isolated from 53 donors (25 female, 28 male; 13 to 80 years old).	Highly clonogenic BM-MSCs were more frequent in preparations from younger female donors.	[50]
	BM-MSCs isolated from 17 donors (25 to 81 years old).	BM-MSCs from young donors showed improved glycosaminoglycan deposition and increased expression of the chondrogenic markers *SOX9*, *COL2A1*, and *ACAN*.	[49]
	BM-MSCs isolated from donors with primary osteoarthritis, osteoporosis, and healthy donors.	BM-MSCs from patients produced chondrogenic pellets of reduced diameter.	[51]
	BM-MSCs isolated from donors with advanced osteoarthritis and healthy donors.	BM-MSCs from patients had a reduced proliferative capacity and a significant reduction in in vitro chondrogenic activity.	[52]
Tissue	AD-MSCs and BM-MSCs isolated from the same donor.	Collagen II and proteoglycans were synthesized only in the BM-MSCs in vitro.	[54]
	AD-MSCs, BM-MSCs, and MSCs from periosteum isolated from the same donor.	Bone marrow and periosteum yielded more homogenous MSCs than fat, improving the correction of physeal arrest in a rabbit model.	[55]
	BM-MSCs and AD-MSCs seeded onto two different scaffolds: Chondro-Gide or Alpha Chondro Shield.	Chondro-Gide seeded with BM-MSCs had the highest MSC proliferation and deposition of ECM tissue.	[56]
	BM-MSCs and AD-MSCs in a platelet-rich plasma scaffold in an osteochondral defect rabbit model.	BM-MSCs demonstrated improved morphological, histological, and immunohistochemical characteristics, higher cartilage-specific gene and protein expression, as well as subchondral bone regeneration.	[57]
	BM-MSCs, AD-MSCs, and cartilage-derived MSCs from adult Sprague Dawley rats.	AD-MSCs have the highest proliferation potential according to growth curve, cell cycle, and telomerase activity analyses.	[58]
	BM-MSCs, AD-MSCs, and UCB-MSCs.	UCB-MSCs could be cultured the longest and showed the highest proliferation capacity.	[27]
	Equine-derived BM-MSCs and UCB-MSCs.	BM-MSCs synthesized ECM of higher quality with a more homogenous distribution of type IIB collagen.	[59]
	SM-MSCs, AD-MSCs, and BM-MSCs isolated from the same donor.	SM-MSCs had the greatest potential for both proliferation and chondrogenesis.	[28]
Tissue location	BM-MSCs isolated from the iliac crest, vertebral body, and femoral head.	BM-MSCs from the iliac crest and vertebral body demonstrated higher chondrogenic potential.	[61]
	BM-MSCs isolated from femur trabeculae through rasping and from the main marrow compartment.	BM-MSCs from femur trabeculae displayed increased chondrogenic potential.	[62]
	AD-MSCs from superficial subcutaneous, deep subcutaneous, omentum, mesentery, and retroperitoneum.	AD-MSCs from subcutaneous tissue show increased proliferative ability and a higher level of CD146 expression.	[63]
	Donor-matched AD-MSCs from knee infrapatellar and subcutaneous adipose tissue of osteoarthritic donors.	AD-MSCs from the infrapatellar fat pad demonstrated increased glycosaminoglycan production and upregulation of the chondrogenic genes *ACAN* and *COL2A1*.	[64]
Subpopulation	Single-cell RNA sequencing of human primary Wharton’s jelly-derived MSCs from three donors.	Differentially expressed gene analysis found several distinct subpopulations of MSCs that differ in proliferation, development, and inflammation response.	[66]
	Single-cell RNA sequencing of BM-MSCs (three donors), AD-MSCs (three donors), UCB-MSCs (two donors), and dermis-derived MSCs (three donors).	MSC subpopulations were substantially heterogeneous in immune regulation, antigen processing/presentation, and senescence.	[65]

## Data Availability

Not applicable.

## Abbreviations

3D	three-dimensional
ACI	autologous chondrocyte implantation
AD-MSC	adipose-derived mesenchymal stem cell
BM-MSC	bone marrow mesenchymal stem cell
ECM	extracellular matrix
EGF	epidermal growth factor
FACS	fluorescence-activated cell sorting
FGF2	fibroblast growth factor 2
GSTT1	glutathione S-transferase theta 1
IGF	insulin-like growth factor
M-ACI	matrix-induced autologous cartilage implantation
MACS	magnetic-activated cell sorting
MALBAC	multiple annealing and looping-based amplification cycles
MDA	multiple displacement amplification
MSC	mesenchymal stem cell
OATS	osteochondral autograft transfer system
PDGF-BB	platelet-derived growth factor-BB
scRNA-seq	single-cell RNA sequencing
SM-MSC	synovium membrane-derived mesenchymal stem cell
UCB-MSC	umbilical cord blood-derived mesenchymal stem cell
